# Boron Induces Lymphocyte Proliferation and Modulates the Priming Effects of Lipopolysaccharide on Macrophages

**DOI:** 10.1371/journal.pone.0150607

**Published:** 2016-03-02

**Authors:** Indusmita Routray, Shakir Ali

**Affiliations:** Department of Biochemistry, Faculty of Science, Jamia Hamdard Deemed University, Hamdard Nagar, New Delhi, 110062, India; Istituto Superiore di Sanità, ITALY

## Abstract

Chemical mediators of inflammation (CMI) are important in host defense against infection. The reduced capacity of host to induce the secretion of these mediators following infection is one of the factors in host susceptibility to infection. Boron, which has been suggested for its role in infection, is reported in this study to increase lymphocyte proliferation and the secretion of CMI by the lipopolysaccharide (LPS)-stimulated peritoneal macrophages in BALB/c mice. Boron was administered to mice orally as borax at different doses for 10 consecutive days, followed by the stimulation of animals with ovalbumin and isolation of splenocytes for proliferation assay. The lymphocyte subsets were determined by flow cytometry in spleen cell suspension. The mediators of inflammation, TNF-α, IL-6, IL-1β and nitric oxide (NO), were measured in culture supernatant of LPS-primed macrophages isolated from borax treated mice. TNF and ILs were measured by ELISA. NO was determined by Griess test. The expression of inducible nitric oxide synthase (iNOS) in macrophages was studied by confocal microscopy. Results showed a significant increase in T and B cell populations, as indicated by an increase in CD4 and CD19, but not CD8, cells. Boron further stimulated the secretion of TNF-α, IL-6, IL-1β, NO and the expression of iNOS by the LPS-primed macrophages. The effect was dose dependent and most significant at a dose level of 4.6 mg/kg b. wt. Taken together, the study concludes that boron at physiological concentration induces lymphocyte proliferation and increases the synthesis and secretion of pro-inflammatory mediators by the LPS-primed macrophages, more specifically the M1 macrophages, possibly acting through Toll-like receptor. The study implicates boron as a regulator of the immune and inflammatory reactions and macrophage polarization, thus playing an important role in augmenting host defense against infection, with possible role in cancer and other diseases.

## Introduction

The chemical mediators of inflammation (CMI) are released by the monocytes and macrophages activated by microbial signals, including the lipopolysaccharide (LPS). These mediators, particularly the tumor necrosis factor α (TNF-α), interleukin (IL)-1β, IL-6, and nitric oxide (NO) have been implicated in host defense against infections [1–4]. Decreased ability of the cells of our immune system to induce rapid increase in activity of CMI following an infection has been reported to cause elevated susceptibility of host to infections [2].

Boron is a naturally occurring element, representing 0.001% of Earth’s crust. It is a metalloid that typically occurs in nature as borate (Na_2_B_4_O_7_) hydrated with varying amount of water [5]. Sodium borate, which is generally described as sodium tetraborate decahydrate (Na_2_B_4_O_7_⋅10H_2_O) and commonly known as borax, is an important compound of boron and a salt of boric acid (H_3_BO_3_) [6]. Boron is present at low concentrations in animal and human. The normal levels of boron in soft tissues, urine and blood usually range from <0.05 ppm to no more than 10 ppm [7]. In animals and human, boron has been implicated in hormone and mineral metabolism and is also reported for its ability to modulate the inflammatory response [8–13]. In postmenopausal women, for example, daily supplementation of 3 mg boron is reported to induce changes consistent with the prevention of Ca^2+^ loss and bone demineralization [13]. In another study, the estimated incidence of arthritis, which is an inflammation of one or more joints, was found to be low (0–10%) in the areas of the world where boron intake was 3–10 mg/day; against 20–70% in areas where the intake was 1.0 mg or less, indicating the role of boron in chronic inflammatory disease [12]. In BALB/c mice, low dietary boron is reported to alter the cytokine profile and affect inflammatory response and survival of *Heligmosomoides bakeri*, a nematode [9]. Boron has also been reported to stimulate and potentiate the release of angiogenesis factor and the heat shock protein (HSP) 70—both of which are linked with inflammation [14–15]. Recently, boron has been implicated in ameliorating the changes associated with hepatocellular carcinoma [16]. Organic compounds containing boron, such as boromycin, a polyether-macrolide antibiotic isolated from *Streptomyces*, have been reported in nature [17–18]. Boromycin inhibits the replication of both clinically isolated as well as cultured strains of HIV-1 [18]. In human, a sodium borate transporter, encoded by *SLC4A11*, reportedly regulating the intracellular boron concentration has been identified, indicating the role of boron in human physiology [19]. In this study, we envisaged and reported the role of boron on the cells of the immune system in BALB/c mice, particularly its effect on non-specific (innate) immune response.

## Materials and Methods

### Reagents

The sources of chemicals and biologicals used in this study were as follows: Dulbecco's Modified Eagle Medium: Nutrient Mixture F-12 (DMEM/F12) (HiMedia, Bombay, India); Fetal Bovine Serum (FBS) (Sigma-Aldrich. Co., USA); Concanavalin A (ConA) (Sigma-Aldrich Co., USA); Lipopolysaccharide (LPS) (Sigma-Aldrich Co., USA); Chicken egg albumin/ovalbumin (OVA) (Sigma-Aldrich Co., USA); Dimethylsulfoxide (DMSO) (SRL, India); 3-(4,5-Dimethylthiazol-2-yl)-2,5-diphenyltetrazolium bromide (MTT) (SRL India); Trypan blue (HiMedia, Bombay, India); 96- and 24-well flat bottom cell culturing plates (Tarson, India); Antibiotic-antimycotic solution (Gibco, USA); ELISA kits for TNF-α, IL-1β and IL-6 (e-Bioscience, San Diego, CA); Fluorescent antibodies and stain buffer (BD Biosciences, USA); Primary antibody against inducible Nitric Oxide Synthase (iNOS) (Santacruz, USA); Alexa Fluor^®^ 488 secondary antibody (Gibco, USA); Vectashield Mounting Medium (VMM) with 4',6-diamidino-2-phenylindole (DAPI) (Vector Laboratories, USA). All other chemicals and reagents used in this study were of analytical grade of highest purity, purchased from local suppliers.

### Animals and Ethics Statement

BALB/c mice (18–22 g, male) procured from the central animal house facility of the University were used in the study. Mice were housed in polypropylene cages and maintained at 21 ± 2°C and >40% humidity. The animals were exposed to a photoperiod of 12 h and had free access to pellet diet and water *ad libitum*. The study was approved by the animal ethics committee of the institute and performed according to the guidelines of CPCSEA (Committee for the Purpose of Control and Supervision of Experiments on Animals, Govt. of India) for the care and use of laboratory animals.

### Lymphocyte Proliferation Assay: Treatment Protocol and Procedure

The lymphocyte proliferation was determined by the MTT assay according to the method described by Huang et al for mouse splenocytes [20]. This method has been used by others [21–25]. Briefly, animals were divided into five groups, each containing six mice. In the boron treated groups, animals were orally administered with different doses of sodium tetraborate decahydrate, viz., 3, 4 and 4.6 mg/kg body weight (b. wt.), corresponding to 2, 2.5 and 3 mM borax, dissolved in double distilled water, for 10 consecutive days. After the completion of the treatment with borax, prime (first) dose of OVA (emulsified in Complete Freund's Adjuvant) was administered intramuscularly on day 11 at a dose level of 50 μg per mouse. The booster dose (OVA, 25 μg per mouse, prepared in Incomplete Freund's Adjuvant) was injected 8 days after administering the prime dose, as described in literature [26–28]. Animals were sacrificed 12 days after the administration of the first dose of OVA. The animals in the control group (C1) did not receive any treatment. The group of animals that received OVA as per the treatment protocol described above but did not receive boron served as positive control (C2). The groups of animals treated with 3, 4 and 4.6 mg/kg borax and challenged with OVA were called as B1, B2 and B3 groups, respectively.

The animals sacrificed after the completion of the treatment protocol were used to harvest the spleen in aseptic condition. The spleen was used to prepare a single cell suspension of spleen cells in DMEM-F12. Erythrocytes were removed by lysis in the presence of 0.9% NH_4_Cl. After the lysis of red cells, the sample was re-suspended in complete DMEM-F12 containing 10% fetal bovine serum and 1% antibiotic-antimycotic solution. Cell viability was tested using the trypan blue exclusion method. Viable cells were cultured in a 96-well culture plate at a density of 2 × 10^4^ cells/well and incubated with 200 μl of OVA (5 μg/ml) or ConA (2 μg/ml) in the presence of CO_2_ (5%) at 37°C for 72 h, and then incubated for another 1 h with MTT (20 μl, 5 mg/ml), prepared in phosphate buffer saline (PBS), pH 7.4 [20]. Spleen cells in suspension consist of a mixture of adherent and non-adherent cells [29]. In the method described above, cells were incubated for 72 h, which resulted in the maximum number of cells settling down. After another one hour of incubation with MTT, and the formation of MTT formazan, the medium was carefully aspirated out without disturbing the settled cells. The formazan crystals were dissolved in 200 μl DMSO and the suspension was kept in dark at room temperature for 2 h till a purple color developed. The absorbance of the purple color was taken at 570 nm in an ELISA reader. The splenocyte culture in plain medium (which did not contain OVA or ConA) was used as negative control.

### Collection of Peritoneal Macrophages and Analysis of iNOS and NO

The peritoneal macrophages were isolated from animals treated with different doses of borax for 10 consecutive days. The macrophages were isolated after a day of completion of the treatment (with borax) as per the published protocol [30]. The isolated macrophages were seeded onto a coverslip in a 24-well flat bottom plate at a density of 1 × 10^6^ cells per ml in 500 μl media and stimulated with LPS (1 ng/ml) for 24 h [31].

For the expression analysis of iNOS, the macrophages were fixed in 4% paraformaldehyde and permeabilized with methanol followed by blocking with 3% BSA. The treated macrophages were stained with mouse anti-iNOS monoclonal antibody (1:100) overnight at 4°C. After the incubation, coverslip was washed with PBS and incubated again at room temperature with Alexa Fluor^®^ 488 rabbit anti-mouse (1:200) (Gibco) for 2 h. The coverslip was finally washed with PBS and mounted using VMM with DAPI. Images were generated and captured with a LEICA SP5II microscope using a 40x objective. Fluorescence was quantified by imageJ software.

For nitrite assay, the macrophages, isolated as above [30], were washed twice with PBS. The pellet (cells) was suspended in DMEM-F12 containing FBS and antibiotic solution and seeded in a 24-well plate at a density of 1 × 10^6^ cells per ml [32]. The plate was incubated in a humidified atmosphere for 2 h at 37°C in the presence of 5% CO_2_. Non-adherent cells were removed and the plate was washed twice with warm media so that the adherent cells (macrophages) did not leach out [32]. The final volume in each well was made up to 500 μl and then the plate was incubated at 37°C for 24 h in the presence or absence of LPS (1 ng/ml). The plate was centrifuged and the supernatant from each well was stored at -80°C. Nitrite in the supernatant was determined by Griess test [33]. Briefly, the supernatant (100 μl) was mixed with an equal volume of Griess reagent (1% sulphanilamide, 0.1% naphthylethylene diamine and 2.5% H_3_PO_4_) and incubated at room temperature for 10 min in a 96-well plate to allow the chromophore formation. The absorbance of the colored solution was measured at 540 nm in an ELISA reader. The standard calibration curve was prepared using sodium nitrite.

### Estimation of Pro-Inflammatory Cytokines

Briefly, the peritoneal cells isolated as above were stimulated with 1 ng/ml LPS for 24 h. Supernatant was harvested and used for the analysis of TNF-α, IL-6 and IL-1β using an ELISA kit (e-Bioscience, USA) and appropriate antibodies.

### Immune Phenotyping

The spleen (1/3^rd^ of the organ), collected from mouse after completion of 10 days of treatment with borax, was taken in ice-cold buffer (PBS, without Mg^2+^ and Ca^2+^) and used to prepare spleen cells suspension. Erythrocytes in the tissue were removed by lysis with 0.9% NH_4_Cl. The suspension obtained after the lysis of red cells was washed properly (thrice) in FBS and then centrifuged at 300 × *g* for 5 min at 4°C. The spleen cells (1 × 10^6^ cells/ml) were re-suspended in cold stain buffer and incubated with fluorescent anti-CD4 PE, anti-CD8 FITC, or anti-CD19 PE antibodies and placed on ice for 30 min in dark. The unbound antibodies were removed by suspending the cell pellet in 0.5 ml stain buffer. The cells, re-suspended in stain buffer (0.5 ml), were analyzed by a flow cytometer (BD LSR II). CD8-FITC and CD4-PE were analyzed together, while CD19-PE was analyzed in a separate tube.

### Statistical Analysis

Statistical analysis was performed using GraphPad Prism5. Variables were analyzed by one-way ANOVA followed by Bonferroni correction. Mean ± S.E.M. of six mice (n = 6) was taken in each set of experiment; *p*<0.05 was taken as statistically significant.

## Results

### Boron and Lymphocyte Proliferation

**Fig 1:**
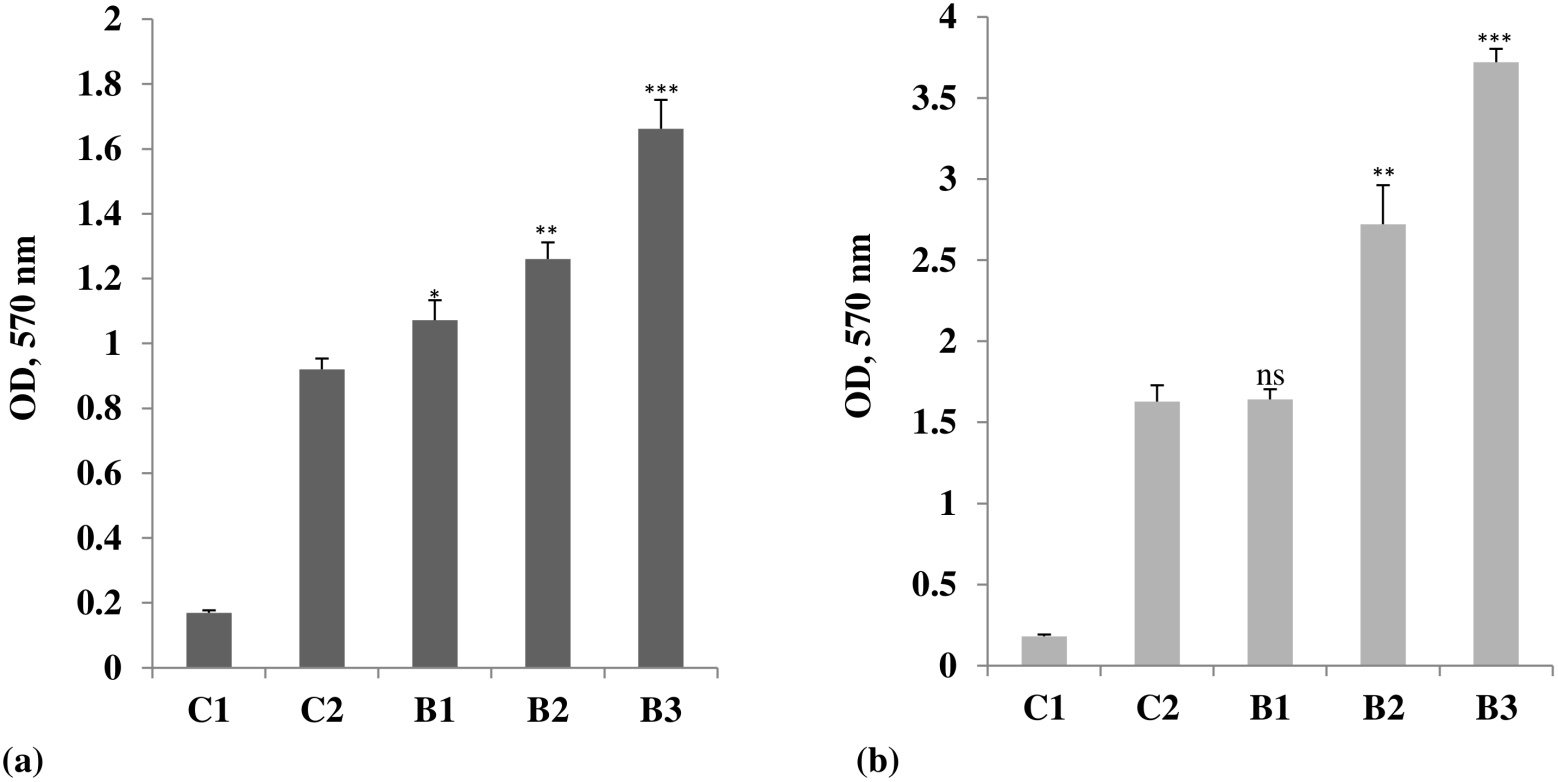
Lymphocyte proliferation in borax treated mice stimulated with (a) Ovalbumin and (b) Concanavalin A. Treatment protocol and other experimental details are same as described in the text. Briefly, the control group of animals (C1) did not receive any treatment. C2, on the other hand, received ovalbumin, but not borax, as per the treatment protocol described in the Materials and Methods section of the text. B1, B2 and B3 indicate different doses of borax administered to mice for 10 consecutive days. Boron increased the lymphocyte proliferative responses of (a) Ovalbumin and (b) Concanavalin A, indicating the B and T cells activation. *p<0.05, **p<0.01 and ***p<0.001, when compared with the control groups; ns = non-significant. The effect of borax was maximum at B3, that is, at a dose level of 4.6 mg/kg b. wt.

The lymphocyte proliferation assay was performed in order to assess the general effect of boron on proliferation of lymphocytes in response to an antigen, OVA, and a mitogen, ConA. The proliferation of the splenocytes was measured by following the reduction of MTT. Boron significantly increased the lymphocyte proliferative response of OVA and ConA. It caused a dose-dependent increase in the antigen (OVA)-induced proliferation of the splenocytes, measured in terms of change in absorbance at 570 nm, showing a maximum increase (1.66 ± 0.08) at a dose of 4.6 mg/kg b. wt. borax, when compared with the stimulated group (0.92 ± 0.03). The value in untreated and unstimulated control group was 0.168 ± 0.008 (Fig 1a). The ConA-stimulated cell proliferation also registered an increase in mice treated with borax. The proliferation measured in terms of the change in absorbance at 570 nm was maximum (3.72 ± 0.10) at 4.6 mg/kg b. wt., when compared with the control groups. The absorbance in stimulated group was 1.63 ± 0.13, while in unstimulated group, it was 0.18 ± 0.012 (Fig 1b).

### iNOS and Nitrite in Boron Treated Mice

**Fig 2:**
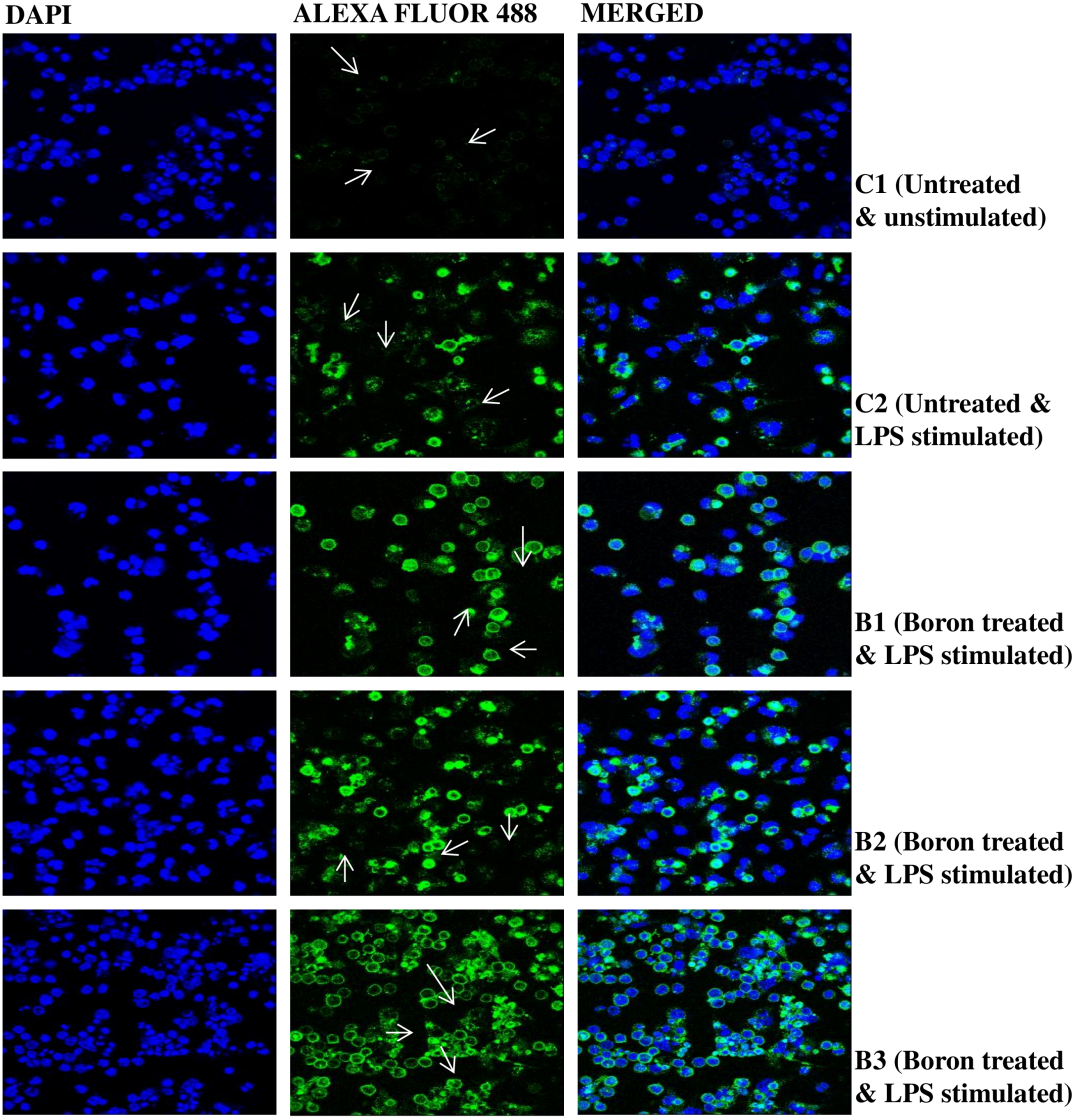
Confocal microscopic images of inducible nitric oxide synthase (iNOS) in peritoneal macrophages isolated from borax treated mice. Mice were treated with different doses of borax, B1, B2 and B3, as described in Materials and Methods section of the text. The isolated macrophages were incubated with primary iNOS antibody followed by incubation with Alexa Fluor^®^ 488. Images were captured in a LEICA SP5II confocal microscope at 40x (magnification). Arrows indicate increase in fluorescence of iNOS conjugated with Alexa Fluor^®^ 488. DAPI: 4',6-Diamidino-2-phenylindole. In all groups, except the untreated and unstimulated control group, cells were stimulated with lipopolysaccharide (LPS) after isolation from mouse peritoneum.

**Fig 3:**
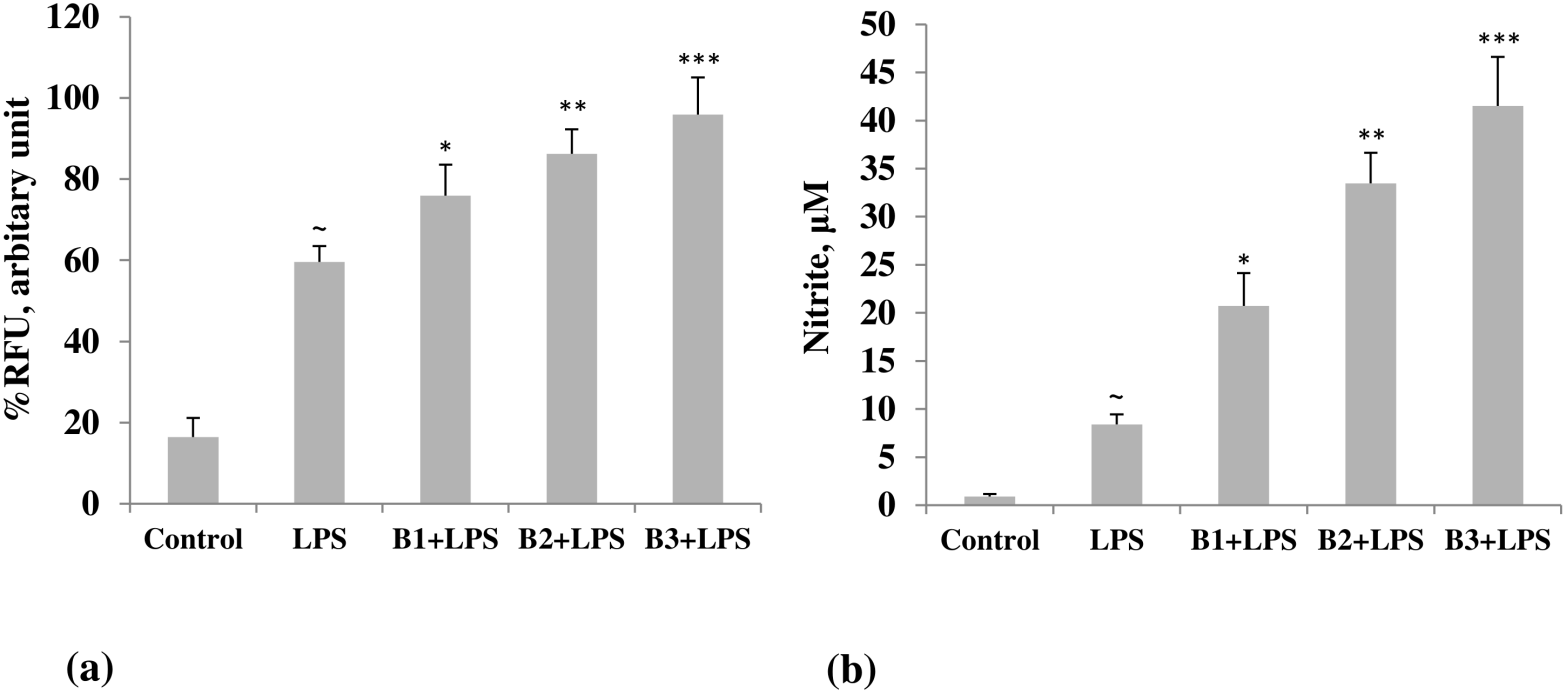
Quantification of (a) iNOS and (b) NO in borax treated and control groups of mice. Mice treated with different doses of borax, as described in the text, were used to isolate the macrophages. Macrophages were cultured and primed with lipopolysaccharide (LPS, 1 ng/ml) for 24 h. The images of iNOS, captured by LEICA SP5II, were quantified using imageJ. NO (nitric oxide) was estimated as nitrite in the supernatant by Griess test. *p<0.05, **p<0.01 and ***p<0.001 indicate significant differences when borax treated groups were compared with LPS-stimulated group. There was a significant (^∼^) increase in both iNOS and NO by the LPS primed macrophages when compared with unstimulated (control) group. B1, B2 and B3 indicate different doses of borax, as described in the text. The maximum effect was seen in mice treated with 4.6 mg/kg b. wt. borax (B3).

iNOS, the inducible nitric oxide synthase, as determined by confocal microscopy, indicated a marked increase in expression in LPS-primed macrophages in borax treated mice. The effect was dose-dependent, showing a maximum increase at 4.6 mg/kg b. wt. of borax (Fig 2). The image intensity, which was quantified by imageJ, was considerably low in both LPS-stimulated and unstimulated control groups (Fig 3a). Increase in expression of iNOS was correlated with an increase in NO, which was measured as nitrite by Griess test in the corresponding groups. Nitric oxide is a mediator in both acute and chronic inflammations. Boron caused a significant increase in the synthesis of nitrite by the LPS-primed macrophages in a dose-dependent manner with a maximum increase (41.52 ± 5.08 μM) at 4.6 mg/kg b. wt. of borax (Fig 3b). In the group stimulated with LPS alone, but not treated with boron, the concentration of nitrite was 8.38 ± 1.05 μM. In the unstimulated control group, it was 0.89 ± 0.28 μM.

### Pro-Inflammatory Cytokines and Boron

**Fig 4:**
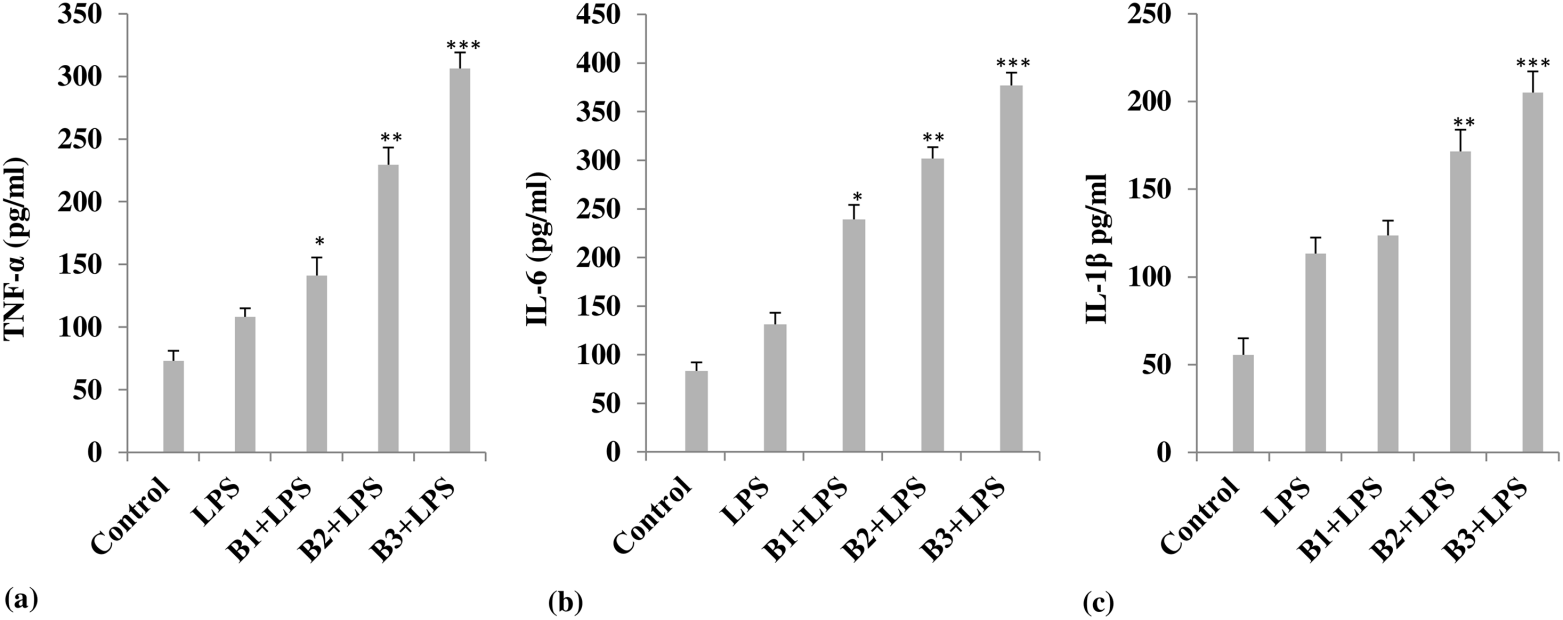
Effect of borax on the release of (a) TNF-α, (b) IL-1β and (c) IL-6 by the lipopolysaccharide (LPS)-primed macrophages. Each value in bar diagrams represents Mean ± S.E.M. (n = 6); *p<0.05, **p<0.01 and ***p<0.001, when compared with LPS-stimulated group. Treatment with borax caused a substantial increase in all three cytokines when compared with LPS stimulated group. Maximum activity was shown at a dose level of 4.6 mg/kg b. wt. of borax (B3). B1, B2 and B3 indicate different doses of borax, as described in the text. In all groups, except control, macrophages were challenged with LPS.

Borax treatment increased the secretion of pro-inflammatory cytokines by the mouse peritoneal macrophages stimulated with 1 ng/ml LPS (Fig 4). Boron increased the secretion of TNF-α, IL-1β and IL-6 by the LPS-primed macrophages. At a concentration of 4.6 mg/kg b. wt. of borax, TNF-α increased to 306.34 ± 12.78 pg/ml, when compared with LPS alone stimulated group (108.06 ± 6.96 pg/ml) (Fig 4a). In unstimulated and untreated control, TNF-α was 73.01 ± 8.0 pg/ml. IL-6 and IL-1β also showed a similar dose-dependent increase in secretion by the peritoneal macrophages isolated from borax treated mice and stimulated with LPS, with a maximum increase shown at 4.6 mg/kg b. wt. of borax. At this dose level, IL-6 increased from 131.11 ± 12.09 pg/ml in LPS alone-stimulated group to 377.07 ± 13.22 pg/ml in group treated with borax and also stimulated with LPS. In the control group (untreated and unstimulated), IL-6 was 83.34 ± 8.86 pg/ml (Fig 4b). The IL-1β level in LPS-primed macrophage culture supernatant of borax treated mice was 205.08 ± 12.02 pg/ml, when compared with the LPS alone-stimulated (113.36 ± 9.02 pg/ml) and untreated/unstimulated control (55.48 ± 9.48 pg/ml) groups (Fig 4c).

### Effect of Boron on Immune Phenotyping

**Fig 5:**
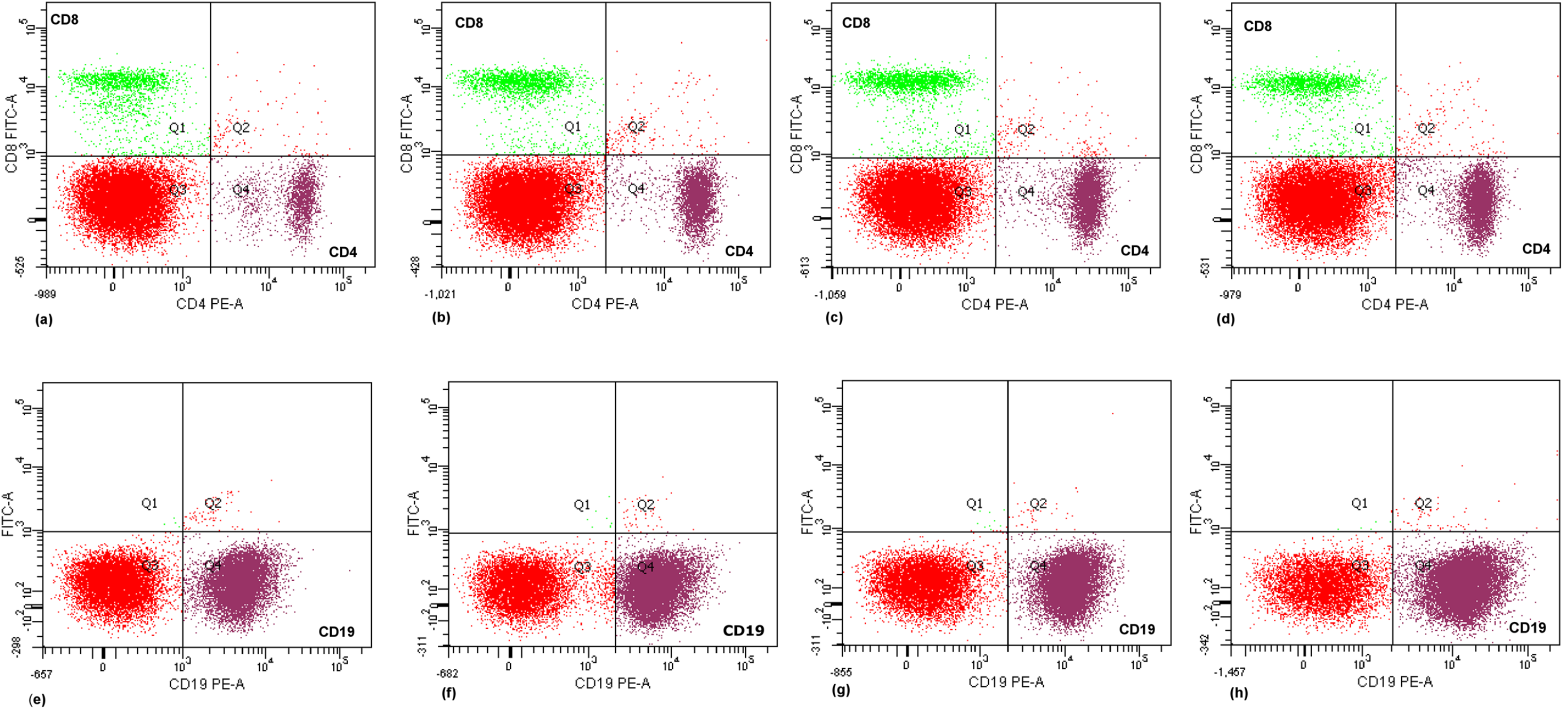
Flow cytometric analysis of B and T cell subsets in borax treated mice. The splenocytes in suspension were labeled with FITC-conjugated CD8 and PE-conjugated CD19 and CD4 antibodies and analyzed as per the protocol described in methods section. Plots (a) and (e) show the percent population of CD8, CD4 and CD19 cells in untreated control group, which was 8.40 ± 0.65, 9.80 ± 1.65 and 50.40 ± 1.9, respectively. Plots (b), (c) and (d) indicate the percent population of CD8 and CD4 cells in borax treated mice. Plots (f), (g) and (h) show the percent population of CD19 cells. The percent population of CD19 and CD4 cells increased in borax treated mice. Population of CD19 cells increased from 50.40 ± 1.9 to 60.65 ± 1.2, 63.20 ± 2.4 and 66.10 ± 1.2 at different doses of borax, with maximum increase at 4.6 mg/kg b. wt. Percent population of CD4 cells in the suspension was 15.2 ± 1.21, 18.3 ± 0.85 and 20.1 ±1.11 in borax treated groups when compared with the respective control group, Plot (a). CD8 cells did not show a significant change in any of the treatment groups.

As mentioned in materials and methods section, cells in suspension were labeled with FITC-conjugated CD8 and PE-conjugated CD19 and CD4 antibodies and analyzed separately by flow cytometry (Fig 5). Boron caused a significant (*p*<0.05) and dose-dependent increase in CD19 (B) and CD4 (T) cell subsets (Table 1). There was a more than two-fold increase in CD4 cells when compared with untreated control. The percentage population of T cell subset CD8 did not show much variation in different groups. In control groups, the percentage of CD19, CD4 and CD8 cells was 50.40 ± (1.9), 9.80 (±1.65) and 8.40 (±0.65), respectively. CD19 and CD4 cell populations increased significantly in groups that received the borax treatment. Maximum increase in B (CD19) and T (CD4) subsets was recorded at a dose of 4.6 mg/kg b. wt borax.

**Table 1:** Percentage population of CD19, CD4 and CD8 cells in boron treated and untreated groups.

Group	CD19	CD4	CD8
Untreated control	50.40 ± 1.9	9.80 ± 1.65	8.40 ± 0.65
Borax (2 mM)	60.65 ± 1.2^a^	15.2 ± 1.21^a^	9.80 ± 0.45
Borax (2.5 mM)	63.20 ± 2.4^b^	18.3 ± 0.85^b^	9.90 ± 0.83
Borax (3 mM)	66.10 ± 1.2^c^	20.1 ± 1.11^c^	10.2 ± 0.68

Boron caused a significant (^a^ *p*<0.05, ^b^ *p*<0.01 and ^c^ *p*<0.001) and dose-dependent increase in CD19 (B) and CD4 (T) cell populations, as measured by flow cytometry. The T cell subset CD8 did not show much variation in any of the treated groups.

## Discussion

Chemical mediators of inflammation secreted by the host monocytes and macrophages constitute an important aspect of systemic host response to infection and diseases such as cancer [1–3,34]. Decrease in the ability of susceptible (CBA/Ca) mice to induce rapid TNF activity following pneumococcal infection, for example, increases the susceptibility of these mice to infection, when compared with the resistant (BALB/c) strain [1]. In our study, we provided evidence suggesting the immune priming effect of borax, a salt of boric acid, H_3_BO_3_, in BALB/c mice. Boron, which has been implicated in infection and immunity [9,11,35–36], is reported in this study to induce lymphocyte proliferation and increase the release of inflammatory mediators (cytokines and NO) by the peritoneal macrophages stimulated with LPS.

Induction of adaptive immune response depends upon the presence of a cognate antigen and antigen specificity of the receptor on the lymphocyte surface, as well as on the presence of pro-inflammatory cytokines such as TNF-α, IL-6 and IL-1 [37–43]. Lymphocyte proliferation is a process in which lymphocytes rapidly increase in number in response to an antigen or stimulation by a mitogen. The dose-dependent increase in B and T lymphocytes observed in this study in splenocytes isolated from borax treated mice and challenged with an antigen and a mitogen indicated the B and T cells proliferation by boron. B and T cells are two broad classes (subtypes) of lymphocytes. All kinds of lymphocytes start in the bone marrow, but while the B subtype remains in the marrow before entering into the bloodstream, T subtype travels to the thymus and mature there in a specific environment. OVA induces the proliferation of B cells which, unlike the other lymphocyte subtypes (T cells and natural killer cells), express receptors to facilitate the binding of antigen (OVA) against which B cell produces antibody [40]. ConA, on the other hand, triggers T lymphocyte by interacting with the receptor on T cell [37]. Direct activation of the receptor on the lymphocyte surface, therefore, plays an important role in the induction of adaptive immune response, beside other factors, including the macrophages, which have been reported to participate in the activation of the lymphocytes.

Boron has an unusual chemistry which has been suggested to help stabilize the structures of various macromolecular complexes including the membrane and protein complexes [44–45]. As suggested by Hunt, boron may interact with one-to-four OH groups or a nitrogen group on specific biological ligands, such as the flavin/pyridine nucleotides and serine proteases. It can interact with the ribosyl moiety of the nucleotide via *cis*-OH or, in case of serine proteases, involving imidazole group of histidine via a nitrogen or OH group on serine [44,46]. The inhibition of the activity of enzymes which require flavin/pyridine nucleotides is reported to be reversible, suggesting that boron might be a regulator of the activity of these enzymes, thereby controlling the relevant cell function including the respiratory burst, which is an important host defense against infectious particles [44]. The boron atom in bortezomib, an FDA approved drug in multiple myeloma, is suggested to reversibly bind to the catalytic site of the 26S proteasome which cancer cells need to survive and multiply [47]. These findings led us to postulate that boron might help proliferative the lymphocytes by stabilizing the antigen-receptor complex on lymphocyte surface, thus causing an augmentation in response. Boron can similarly stabilize the structure of lymphocyte enhancer factor-1, a 48 kD nuclear protein in pre-T and B lymphocytes, which has been implicated in Wnt signaling and lymphocyte proliferation [48]. Other complexes such as the LPS-CD14/TLR4/MD2 complex, which induces the synthesis and secretion of pro-inflammatory cytokines by the LPS-primed macrophages, more specifically by M1 macrophage, may also be stabilized by boron in a similar manner.

Another mechanistic explanation of the effect of boron on lymphocyte proliferation may be the antioxidant potential of boron compounds. Boron may act by counteracting the inhibitory effect of oxidative stress on lymphocyte proliferation. Oxidative stress has been reported to prevent the transition from the G0 to the G1 phase of the cell cycle and inhibit the proliferative response of human lymphocyte to phytohaemagglutinin [49]. The effect, which could be prevented by 2-mercaptoethanol, a thiol, suggests a critical role of antioxidants in counteracting the effect of oxidative stress on lymphocyte proliferation. The oxidative stress markers have been identified in bronchoalveolar lavage fluid and lung tissue from OVA sensitized mice [50]. Similarly, ConA has been reported to induce a significant increase in lipid peroxidation accompanied by a significant depletion of glutathione and total antioxidant capacity [51]. Glutathione, a thiol, is widely known for its protective action against oxidative stress [52]. There are reports in literature which suggest the antioxidant role of boron [53]. The effect of boron on lymphocyte proliferation, therefore, may be attributed, at least in part, to its ability to counteract the inhibitory effect of oxidative stress on lymphocyte proliferation produced by OVA or ConA. Boron might help the lymphocytes to proliferate by augmenting the antioxidant potential of the cell. In earlier studies, substances like mercury and 1,25-dihydroxyvitamin D3 have been reported to enhance OVA and ConA-induced proliferation of the lymphocytes [43,54].

The antigen, OVA, can stimulate the helper (CD4) and cytotoxic (CD8) T cell subsets in actively immunized animals [41–43]. CD4 cells are particularly involved in sending signals to other cells of the immune system, including the CD8 cells, which then destroy the infected (particularly the virus infected) cells and other damaged cells, including the cancer cells. In this study, which was done in uninfected animals, we did not report an increase in CD8 cells in boron treated mice. CD8 is a cell surface glycoprotein best known for its effects on suppressor/cytotoxic functions and on NK cells [55–56]. It has been associated with the killing of parasites, such as *Leishmania* major, where, acting as a co-receptor, it signals the macrophage function and induces iNOS/NO [57]. Unlike CD8 cells, the population of CD4 cells, however, showed a significant increase in mice treated with borax. The increase in CD4 cells, as reported in this study, suggests an enhanced Th response, eventually promoting the functions of other cells such as the macrophages, which constitute a major component of the immune system in fighting infection and which have been used in this study to investigate the immune priming effect of borax. An increase in percentage population of the CD4 cells has been reported earlier by a marine oligopeptide preparation from chum salmon (*Oncorhynchus keta*), and also by a traditional Chinese medicine, che-shie-shuang-bu-an-shen-tang [58–59]. The medicine significantly increased the number of CD4 cells, but did not affect the proportion of CD8 cells.

CD4^+^T cells, along with CD8^+^T cells, constitute majority of T cells. Proliferation and differentiation of thymocytes (T-cell precursors) into specific effector cells depend upon the T cell receptors (TCRs), which initiate a network of downstream signaling pathways. Lineage-specific differentiation of T cells depends upon the concentration of antigens and costimulatory molecules, among other factors, including the antigen presenting cells [60]. The strength of TCR signal determines the costimulatory requirements for Th1 and Th2 CD4^+^T cell differentiation [61]. Boron, as reported in this study, by virtue of its ability to bind to and stabilize the complex molecular structures, might act as a small molecule to increase the strength of TCR. It can similarly act upon the BCR, the B cell antigen receptor. CD19, which is synthesized by the cells of the B cell lineage, is a hallmark of B cells, the fate and function of which is determined by BCR. CD19 has a regulatory role in proliferation and differentiation of the B cells [62–63]. CD19, in particular, regulates the basal signaling thresholds and accelerates BCR signal [64]. Boron could possibly act by modulating the CD19 expression and/or function, which may explain its effect on the host immune response. Taken together, these findings suggest a possible role of boron as a costimulatory molecule that can augment the strength of T and B cell receptors, thus augmenting the immune response.

As mentioned above, boron caused an increase in CD4 cell population. These cells play an important role in immunity, particularly the adaptive immunity, helping other immune cells by releasing cytokines. CD4 cells are essential in B cell antibody class switching, as well as in the activation and growth of CD8 cells and maximizing the activity of phagocytes, which include the macrophages [65]. Macrophages are the professional phagocytes which patrol our body for potential threat of pathogen. These cells are crucial in non-specific host defense against infection and, beside their role in non-specific or innate immunity, also help initiate the adaptive immune response by recruiting the lymphocytes. Macrophages produce cytokines, which have a number of functions including their role in T and B cell activation and differentiation [66–67]. Macrophages can increase or decrease inflammation, thus regulating the immune response. Pro-inflammatory agents such as LPS have been reported to induce the synthesis of iNOS by the macrophages [68]. The macrophages that promote inflammation and immune response are called M1 macrophages. On the other hand, the macrophages which decrease inflammation and encourage tissue repair are called M2 macrophages. The M1 macrophages, unlike the M2 macrophages, produce NO and other pro-inflammatory cytokines such as the TNF-α, IL-6 and IL-1β to fight infection. iNOS, which is involved in the synthesis of NO, is in fact considered a hallmark of M1 activation. NO biosynthesis through iNOS results in higher concentration of NO for longer duration of time, thus allowing a sustained release of peroxynitrite, a highly reactive nitrogen species produced by the coupling of NO with superoxide anion radical, which is reported to inhibit the growth of many pathogens [69]. The non-activated macrophages also produce NO but in very less amount [70].

NO, besides its direct role as an antimicrobial agent at high concentration, mediates the release of cytokines by the T cell [71]. NO produced in excess, however, disturbs the type 1/type 2 helper T cell balance, resulting in type 2 T cell biased responses. Suppression of the type 1 T helper cell-dependent response has been reported earlier [4]. In our study, we observed an increase in expression of iNOS and a consequent increase in NO in boron treated mice. The level of pro-inflammatory cytokines (TNF-α, IL-6, IL-1β) in culture supernatant of LPS-primed peritoneal macrophages isolated from mice treated with borax also increased. In earlier studies, LPS has been reported to stimulate the secretion of TNF-α, IL-6, IL-1β, and NO by the macrophages with a concomitant increase in expression of iNOS [72]. The increase in pro-inflammatory cytokines and iNOS by the LPS-primed macrophages reported in our study was significantly higher and dose-dependent in the presence of boron when compared with the group stimulated with LPS but not treated with borax, indicating the role of boron as a substance that can potentiate the LPS-induced response of the macrophage. The increase in expression of iNOS, hallmark of the M1 macrophages (which promote inflammation and immune response), as reported in this study, suggests a possible role of boron in macrophage polarization.

The activation of M1 microphages can be induced by the LPS and a variety of signals. LPS stimulates the macrophage by binding to its receptor on the macrophage surface. The LPS receptor belongs to a family of proteins called Toll-like receptor (TLR), more specifically the TLR4, which is dedicated to the detection of microbial signal [73]. LPS binds to the CD14/TLR4/MD2 receptor complex on macrophage surface and promotes the release of pro-inflammatory mediators [74]. Since, as discussed above, the M1 macrophages are linked to pro-inflammatory response, we speculated that the TLR4 pathway might mediate the release of TNF-α, IL-6, IL-1β and NO by the LPS-primed macrophages isolated from borax treated mice. In a recent study, benzoxaborole analog, a boron-containing compound, has been reported to affect (inhibit) the TLR-mediated response, supporting our speculation concerning the involvement of TLR pathway in manifestation of the effect of boron [35]. In another study, boric acid was reported to inhibit the LPS-stimulated secretion of TNF-α [75]. This result, however, is not in agreement with our result—which showed an increase in TNF-α in boron treated mice. The increase observed in our study may be attributed to the presence of glutathione (GSH). In the study by Cao and coworkers [75], the experiment was performed on GSH depleted cells, indicating that the effect of boron on TNF-α involved a thiol-dependent mechanism. The increase in TNF-α has been reported in other studies which include a study in culture supernatant of monocytes isolated from animals on a boron supplemented diet [46]. Boron has also been found to increase the level of TNF-α in cultured human fibroblast and chick embryo cartilage cell [76–77]. It is reported to increase the total mRNA for TNF-α in cultured fibroblast. Increase in TNF-α is crucial for the proliferation of lymphocytes [78].

TNF and ILs affect a variety of cells and induce many similar inflammatory reactions, including the secretion of cytokines, leukocyte adherence, fibroblast activation, chemotaxis, endothelial gene regulation, and fever. In our study, beside TNF-α, we reported an increase in IL-6 and IL-1β. IL-6 can promote or inhibit inflammation. IL-6 stimulates a target cell by a membrane bound receptor which can associate with gp130, a signaling receptor protein which, through a cascade, activates the MAPK pathway [79]. It is worth mentioning that while only few cells express IL-6 receptor, all cells display gp130, and that the cells which only express gp130 are not responsive to IL-6 alone, but can respond to a complex of IL-6 bound to a naturally occurring soluble form of the IL-6 receptor which dramatically enlarges the spectrum of IL-6 target cells in a process called *trans*-signaling [79]. The pro-inflammatory response of IL-6 is reported to be mediated by *trans*-signaling, unlike its (IL-6) regenerative or anti-inflammatory activity, which is mediated by classic signaling [79]. The pro-inflammatory response of the macrophages as reported in our study in LPS stimulated cells (macrophages) isolated from borax treated mice suggests the involvement of *trans*-signaling in boron treated mice. IL-6, which is also called as the B-cell stimulatory factor2, promotes differentiation of B lymphocyte and proliferation of T cells. We also reported an increase in IL-1β in this study. IL-1 binds to its receptors (CD121a/IL1R1, CD121b/IL1R2) which are considerably highly conserved in evolution and can bind to all forms of IL-1. The binding of IL-1 to its receptors is crucial for inflammation and immunity, and also in haematopoiesis. The increase in IL-1β, as reported in our study, therefore, points toward a key role of boron as a regulator of the immune and inflammatory reactions and haematopoiesis. In an earlier study [80], inflammatory challenge has been shown to increase the lymphocyte proliferative response of ConA and increase IL-1β production, which is in agreement with our results. The study reported suppression of the response by fish oil [80]. IL-1β has also been reported to increase the expression of iNOS [81–82], which has been discussed above.

## Conclusion

This study reports the immune priming effects of borax, a salt of boric acid (H_3_BO_3_), in BALB/c mice. Oral administration of borax to mice induced lymphocyte proliferation and increased the synthesis and secretion of pro-inflammatory mediators, cytokines (TNF-α, IL-6, IL-1β) and NO, as well as the expression of iNOS, by the LPS-primed macrophages, more specifically the M1 macrophages, possibly acting through Toll-like receptor. Boron, which has been reported to stabilize the structure of macromolecular complexes, is postulated in this study to act by stabilizing the structures of antigen-receptor complexes involved in lymphocyte proliferation and stimulation of the macrophages to secrete inflammatory mediators as a major mechanism. Besides, boron might help the lymphocytes to proliferate by augmenting the antioxidant potential of the cell. The study implicates boron as a regulator of the immune and inflammatory reactions and macrophage polarization, thus reinforcing its role in augmenting the innate as well as adaptive immunity, with a possible role in cancer and other diseases.
